# A dynamic model of some malaria-transmitting anopheline mosquitoes of the Afrotropical region. II. Validation of species distribution and seasonal variations

**DOI:** 10.1186/1475-2875-12-78

**Published:** 2013-02-25

**Authors:** Torleif M Lunde, Meshesha Balkew, Diriba Korecha, Teshome Gebre-Michael, Fekadu Massebo, Asgeir Sorteberg, Bernt Lindtjørn

**Affiliations:** 1 Centre for International Health, University of Bergen, Bergen, Norway; 2 Bjerknes Centre for Climate Research, University of Bergen/Uni Research, Bergen, Norway; 3 Aklilu Lemma Institute of Pathobiology, Addis Ababa University, Addis Ababa, Ethiopia; 4 National Meteorological Agency of Ethiopia, Addis Ababa, Ethiopia; 5 Arba Minch University, Arba Minch, Ethiopia; 6 Geophysical Institute, University of Bergen, Bergen, Norway

**Keywords:** Anopheles gambiae complex, Model, Malaria

## Abstract

**Background:**

The first part of this study aimed to develop a model for *Anopheles gambiae s.l.* with separate parametrization schemes for *Anopheles gambiae s.s.* and *Anopheles arabiensis*. The characterizations were constructed based on literature from the past decades. This part of the study is focusing on the model’s ability to separate the mean state of the two species of the *An. gambiae* complex in Africa. The model is also evaluated with respect to capturing the temporal variability of *An. arabiensis* in Ethiopia. Before conclusions and guidance based on models can be made, models need to be validated.

**Methods:**

The model used in this paper is described in part one (Malaria Journal 2013, 12:28). For the validation of the model, a data base of 5,935 points on the presence of *An. gambiae s.s.* and *An. arabiensis* was constructed. An additional 992 points were collected on the presence *An. gambiae s.l.*. These data were used to assess if the model could recreate the spatial distribution of the two species. The dataset is made available in the public domain. This is followed by a case study from Madagascar where the model’s ability to recreate the relative fraction of each species is investigated. In the last section the model’s ability to reproduce the temporal variability of *An. arabiensis* in Ethiopia is tested. The model was compared with data from four papers, and one field survey covering two years.

**Results:**

Overall, the model has a realistic representation of seasonal and year to year variability in mosquito densities in Ethiopia. The model is also able to describe the distribution of *An. gambiae s.s.* and *An. arabiensis* in sub-Saharan Africa. This implies this model can be used for seasonal and long term predictions of changes in the burden of malaria. Before models can be used to improving human health, or guide which interventions are to be applied where, there is a need to understand the system of interest. Validation is an important part of this process. It is also found that one of the main mechanisms separating *An. gambiae s.s.* and *An. arabiensis* is the availability of hosts; humans and cattle. Climate play a secondary, but still important, role.

## Background

Several attempts have been made to map the distribution of *Anopheles gambiae s.s.* and *Anopheles arabiensis*[1-5], two of the most important vectors of human malaria in sub-Saharan Africa. MacDonald [6] showed that limiting the human-vector contact reduces malaria transmission, and that the most efficient control measure is to increase the mortality rate of the involved mosquitoes. His thinking has been adopted in current malaria control efforts. Two of the most common interventions today are indoor residual spraying (IRS) [7] and insecticide-treated bed nets (ITNs) [8]. Often, there is no detailed understanding of the life history, behaviour and species composition where the interventions are applied [3].

*Anopheles arabiensis* inhabits areas from South Africa in the south to Mauritania and Sudan in the north. In Central-West Africa there is a pocket with very few observations of *An. arabiensis*. The border of this pocket is formed by Angola, Zambia, Burundi, Rwanda, Uganda, South-Sudan, Central African Republic, Congo, Gabon, and Equatorial Guinea. *Anopheles gambiae s.s.* is currently separated into five chromosomal forms: Forest, Bamako, Savanna, Mopti and Bissau [9], and two molecular forms: M and S [10,11]. It is distributed from South Africa to Mauritania and northern Mali, but is absent in Ethiopia and Northern Sudan. The species is considered the most efficient malaria vector in Africa [12].

Recent studies have shown that interventions aimed to prevent malaria has an impact on balance between *An. gambiae s.s.* and *An. arabiensis*[13]. The relative fraction of each species can vary from month to month, and year to year [14]. In Tanzania it has been shown that multi-decadal changes in the species composition can influence malaria transmission [15]. Given the observed changes in species composition, and their different capacity as vectors of malaria, it is highly relevant to have models which include several species when assessing the impact of climate variability and climate change.

This paper is the second of two describing and validating a new model of the dynamics of *An. gambiae s.s.* and *An. arabiensis* The model, which is described in part one [16], is a biophysical model driven by output from a climate model. Biophysical models seek to understand what drives a certain biological process, and to describe this with mathematical equations. Unlike statistical models, which often rely on observations to predict species presence and absence, biophysical models can be run with no information with respect to observed distribution and densities, and base the model equations on laboratory studies aiming to isolate different aspects of the life history of the mosquitoes. The role of field observations on the presence or absence of a species in the case of biophysical models, is to validate the model after an experiment has been completed. In some studies observations are used to reduce the uncertainty of unknown parameters [17].

In addition to predicting the current distribution, these type of models can be used to project changes in the historical and future density and distribution of these species. They can describe changes from day-to-day, month-to-month, year-to-year, and decade-to-decade. The model, named Open Malaria Warning (OMaWa) [16], includes several components, describing the mosquito’s life from the aquatic stages to adult. In the aquatic stages, life history varies for eggs, larvae and pupae. As adults the life history changes with age. OMaWa is driven with air temperature, relative humidity of the air, wind speed and direction, soil temperature, relative soil moisture, and runoff from a climate model. These variables are used to parametrize mortality, rate at which eggs are laid, biting rate, development rate in the aquatic stages, and dispersion (spread) of mosquitoes. In part one, it was shown how the model responded to different forcings, and focused on its sensitivity to temperature, humidity, mosquito size, the probability of finding blood, and dispersion. Thus the results presented here should be seen in light of the sensitivity analysis. A full description of the model used here can be found in part one [16].

This is the first time a biophysical model has been used to model the relative density of *An. gambiae s.s.* and *An. arabiensis*, with simulations covering an entire continent. It is also the first time age dependent life history and mosquito dispersion (spread of mosquitoes) has been included in a continental analysis. The model is validated against 6,927 presence/absence points of the two species, and a more detailed analysis is carried out for Madagascar. The data is freely available to the public [18]. This study has also evaluated the ability to model the temporal variability, using case studies for Ethiopia.

## Methods

### Occurrence and distribution of *An. gambiae s.l.* in Africa

#### Continental validation

To date there are three data sets describing the occurrence of *An. arabiensis* and *An. gambiae s.s.*[3,19,20]. Additional online resources have been described by Hay *et al*[21]. To compliment and extend these databases, a systematic search was conducted. A total of 1,940 occurrence points were collected for *An. arabiensis*, 1,813 for *An. gambiae s.s.*, and 992 for *An. gambiae*. Merging these data with the three databases [3,19,20] result in 2,926 occurrence points for *An. arabiensis*, 3,009 for *An. gambiae s.s.*, and 992 for *An. gambiae*[18]. Three methods were used to geo-reference the points. In papers where coordinates were given, these coordinates were used. If possible they were cross checked against given place names. In cases where only place name, and a description of the place were given, the locations were searched up using Google Maps/Earth. Where only a map was provided, the map was imported to qgis and geo-referenced [22], and occurrence points were manually extracted.

The database containing *An. gambiae* was mainly used to estimate the occurrence of *An. gambiae s.l.* in Namibia, DRC, South Sudan, Angola, Congo, and northern South Africa. To classify the points the expert opinion polygons from Sinka *et al*[3] was used. A point falling within the *An. arabiensis* polygon only was classified as *An. arabiensis*, points falling within the *An. gambiae s.s.* polygon only as *An. gambiae s.s.*, and points falling within both polygons were assigned both species. To classify true presence/absence points the data described previously was used. Observations of *An. gambiae s.s.* were classified as presence for this species. Absence points for *An. gambiae s.s.* were those where *An. arabiensis* had been recorded, and no *An. gambiae s.s.* had been observed within a radius of 100 km. The same approach was used for *An. arabiensis*.

This model (OMaWa) was compared with species predictions from four other models, as well as the expert opinion from Sinka *et al*[3]. The first was the paper by Rogers *et al*[1] where they used satellite data to predict the presence of *An. arabiensis* and *An. gambiae s.s.*. To reproduce the images in the paper the figures were geo-referenced, and polygons were drawn based on the 0.65-1 probability. The selection was based on the colouring they used in the figure. Next a 50 by 50 km grid was overlaid with the polygons, and points falling within the polygons were classified as presence points. Points falling outside were classified as absence. The second paper is by Levine *et al*[2]. They used a genetic algorithm to predict the presence of the two species. As before, the images were geo-referenced, and polygons were constructed based on dark grey to black shading. Next, absence and presence was constructed as for Rogers *et al*[1]. The third paper is a recent paper by Sinka *et al*[3]. Since this is a three band RGB (Red-Green-Blue) raster, the pixel values were first converted to a one band raster: 1 - (0.299·*R *+ 0.587 · *G *+ 0.114 · *B*)/255. This new raster image was then gridded to a 50 by 50 km grid. Presence was defined as probability greater than approximately 0.4. As for Rogers *et al*[1], this threshold was selected based on the colouring in the figure (and it must be assumed the authors chose the colours based on what they thought to be realistic classifications). Where applicable, the weighted absolute mean error was also calculated based where weights were equal to the probability given in the maps. The fourth paper is by Moffet *et al*[5]. The same methodology as for Sinka *et al*[3] was used to construct a comparable map. For the expert opinion, presence/absence points were constructed with the same methodology used for Levine [2] and Rogers [1]. The extracted data and scripts are available upon request. The mosquito density from OMaWa was classified as present if the 19 year mean was greater than 0.004 mosquitoes per square kilometre, and absent if less. Quality of the models were estimated as mean absolute error (MAE), which is recommended over the root mean square when comparing model performance [23].

#### Relative fraction of each species, Madagascar

To investigate if the model is able to estimate the relative fraction of *An. gambiae s.s.* and *An. arabiensis* data from Pock Tsy’s *et al*[24] and Chauvet’s [25] article describing the fraction of each species in Madagascar was used. In total these two data sets consist of 275 observations, and should thus be suitable to give a rough idea about the relative fraction of the two members. Different measures were given to evaluate the model skill: 

a) For each observation there are information about the month of collection as well as longitude and latitude. From the model data, covering the period 1990-2008, the closest point to each observation in the month of collection is selected, and the yearly monthly mean is calculated. These data were used to make box plots, weighting for the number of observations in each point, comparing the observations with the model. 

b) From the data produced in a, maps were created using a distance weighted kernel with cut off at 100 km. Hence observations further away than 100 km were not included, and closer points will be given more weight. 

c) The distance to the closest wrong (difference in fraction greater than 0.2) and correct (difference smaller than 0.2) prediction will be indexes for the spatial accuracy. A non-parametric test like the Wilcoxon rank sum test with continuity correction (Mann-Whitney) test can then be used to test if the two indexes differ by a location shift of zero, and the alternative is that they differ by some other location shift.

### Temporal variability

#### Model setup

In addition to looking at the spatial patterns, it is of interest how the model reproduce temporal variability in mosquito numbers. Originally, this model was developed to increase the understanding of malaria epidemiology in Ethiopia. The motivation of introducing *An. gambiae s.s.* was to test if the model had a general validity, not limited to Ethiopia. Two high resolution runs only covering Ethiopia were done; one at 30 km, covering the period from 2000 to 2006 (Eth30), and one at 18 km (Eth18) covering the period from 2008 to 2011. These two runs differ from the one covering all of Africa in the way that the weather simulations were forced to follow the observed weather pattern. The technique used to accomplish this is called spectral nudging. In the African run (TC50) the intention was not to reproduce the exact year to year variability, but the interest was to reproduce reasonable weather in a reasonable climate, and thus no nudging was used. To validate the ability to reproduce seasonal variations data from Eth30 and Eth18 to drive OMaWa was used.

For simulations driven by Eth30 the model was run without dispersion, BLL aquatic mortality, development rate with no species correction, default gonotrophic cycle, and AL adult mortality. TC50 and Eth18 were run with the following parametrization: with dispersion, KBLL aquatic mortality, development rate with species correction, default gonotrophic cycle, and BLLad adult mortality. All results are based on single realizations of the model, and error bars are therefore not reported.

#### Validation data

There are few papers describing the year to year, and seasonal variations in mosquito numbers in Ethiopia. In the validation process three papers were used, one master thesis, and field data from Chano Mille, Arba Minch describing mosquito seasonality.

The first, a paper by Kenea *et al*[26], is describing *An. arabiensis* larva density in the vicinity of six villages in central Ethiopia, December 2007 to June 2008. The second paper is by Taye *et al*[27] and is reporting bi-monthly (October 2001 to August 2002) adult *An. arabiensis* numbers in Sille (Southern Ethiopia). The third paper is by Yemane Ye-Ebiyo *et al*[28], where they report larva density in seven naturally formed puddles, in Ziway. Since this paper does not report density in the area as a whole, the data might not be directly comparable to the modelled ones. To overcome this problem all time series were scaled, both observations and model results, as standardized anomalies:

(1) $$\frac{x-\frac{1}{n}\sum x}{\sqrt{\frac{{\left(x-\frac{1}{n}\sum x\right)}^{2}}{n}}}$$

**Figure 1:**
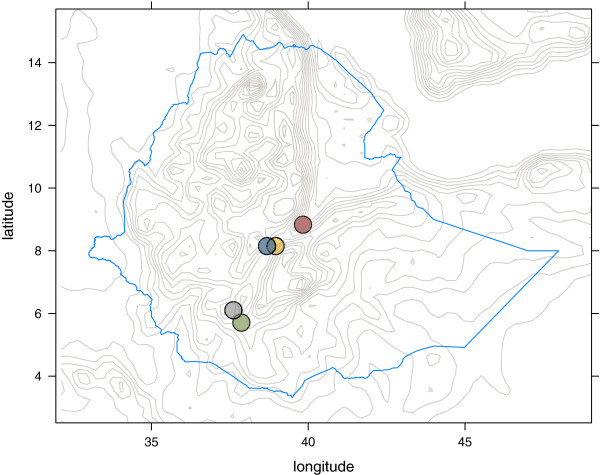
**Areas used for validation of the model. **KEN11 (blue circle), TAY2006 (green circle), YE2003 (orange circle), BAL2001 (red circle), and FEK2012 (grey circle).

To compare the absolute density, it would be required that the papers reported the larva/mosquito density per square kilometre over a larger area. Since this is not the case, scaling is necessary. The last study is by Balkew, where the seasonality of *An. arabiensis* in Awash Valley, Ethiopia, was described [29]. The study locations are plotted in Figure 1.

In addition to the published data, Fekadu Massebo collected one year (May 2009 to April 2010) of mosquito densities in Chano Mille, Ethiopia. The study site is described in [30,31]. To see if the model was able to reproduce the mosquito densities, Eth18 was used to drive OMaWa.

#### Validation statistics

All correlations (Pearson) are calculated from the values reported in the papers [26-29], and a similar time series (sampled the same month as the observations) is constructed from the model averaging the four closest model points:

(2) $$\text{cor}\left(\frac{x_{\text{obs}}-\text{mean}(x_{\text{obs}})}{\text{sd}(x_{\text{obs}})},\frac{x_{\text{mod}}-\text{mean}(x_{\text{mod}})}{\text{sd}(x_{\text{mod}})}\right)$$

### Climate model realizations

The simulations in this paper was driven by three different realizations of a limited area climate model. The first realization (Eth30), carried out in 2009, comes from WRF model version 3.1.1 [32]. It was run at 90 km resolution using a tropical channel set up. In this type of setup, the domain consists of the boundaries above and below certain latitude and no side boundaries. This process allows the interaction from the extra-tropics through the north-and-south boundaries. In addition, it allows the generated waves to propagate around the globe more naturally – as in the real world and in global models. The meridional boundary conditions were specified using six-hourly National Centers for Environmental Prediction (NCEP) Reanalysis 2 (T42) data. The runs have meridional boundaries at 45°*S* and 37°*N*, with 27 vertical levels, ranging from the surface to pressure p = 10 hPa. Inside the channel, a domain with 30 km resolution was set up. This domain has boundaries at 25.56°*E*, 53.18°*E*, 0.24°*N*, and 19.29°*N*. To ensure the model reproduced the observed year to year anomalies, the model was nudged, using spectral nudging, against waves (wind, pressure, and temperature) longer than 1,000 km in both domains. The Kain Frisch cumulus parametrization scheme was used [33,34].

The second realization (TC50), carried out in 2011, had again a tropical channel set up. The model was run at 50 km resolution from January 1 1989 to January 1 2009. At the north and southern boundaries the model was driven by Era Interim. The Kain Frisch cumulus parametrization scheme was used [33,34]. No nudging was used, and therefore it is less probable the model would reproduce year to year variability in the weather. This run was used to assess the mean state of mosquito density and distribution.

In the third experiment (Eth18), done in 2012, WRF 3.3.1 was used with the Tiedtke cumulus parametrization scheme [35,36]. The model was run at 18 km resolution from January 1 2008 to August 1 2011, with data from Era Interim at the boundaries. Outside the planetary boundary layer the same type of spectral nudging as described earlier was applied. The domain had boundaries at 30.57°*N*, 50.99°*N*, 1.45°*S*, and 18.97°*E*.

The Regional Committee for Medical and Health Research Ethics, Western Norway, and the Ethical Committee of the Faculty of Medicine of Addis Ababa University and The National Health Research Ethics Review Committee (NERC) of Ethiopia granted ethical approval for the study.

## Results and discussion

### Distribution of Anopheles gambiae s.l

#### Occurence of Anopheles gambiae s.l. in Africa (TC50)

**Figure 2:**
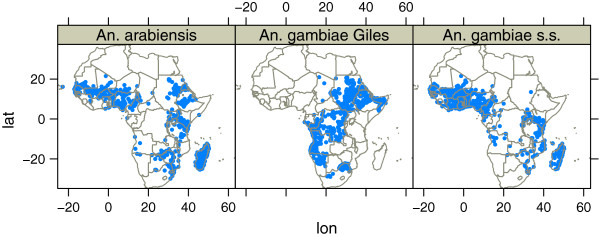
**Presence points for*An. arabiensis*,*An. gambiae s.s. *and*An. gambiae*.**

**Figure 3:**
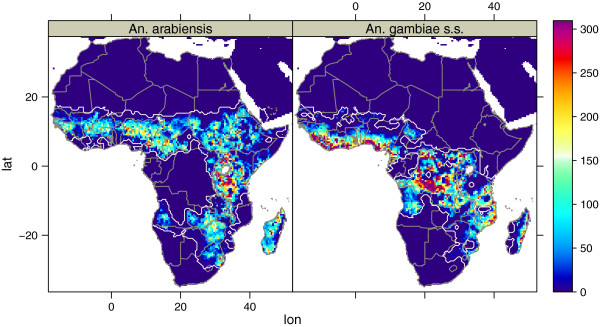
**Mean density of *An. arabiensis * and *An. gambiae s.s. *, 1990-2008. **White contours show where the species were present during the simulation.

Figure 2 is showing the presence data collected as part of this work. Data collection on *An. gambiae* was focused on areas where little information about the occurrence of *An. arabiensis* and *An. gambiae s.s.* was available. Figure 3 shows the modelled mean density of *An. arabiensis* and *An. gambiae s.s.*. The white contours are indicating the presence of each species. The pattern is consistent with the general perception of the species range [3]. This is the first time a model [1-4] has been able to reproduce the absence of *An. gambiae s.s.* in Ethiopia. Still there are some unresolved issues. To date there are no records of *An. arabiensis* in Côte d’Ivoire; no models, this included, have been able to model the absence of this species in Côte d’Ivoire. A look at the figure also reveals some probable inconsistencies with respect to the species distribution in southern Chad where *An. arabiensis* should be dominating [37]. In South-Africa the distribution is consistent with observations from 1958 [38], although the species observed might have been *An. quadriannulatus*. There are however no recent available surveys of *An. gambiae s.l.* in the states of Gauteng, North West or South Western Limpopo. In Namibia, where *An. gambiae s.l.* has been observed as far south as -23.7°*N*[39], the model limits the range to approximately -21°*N*. Since there are no available data on the recent distribution of this complex in Namibia, it is difficult to know whether the model is correct or wrong. The model also suggests *An. gambiae* is absent in large parts of Gabon. Previous studies have found *An. gambiae* in Lambarene [40] and Moyen-Ogooue [41], while Mouchet only found this species in Libreville of twelve sites sampled [42]. It should be noted that Mourou *et al* later found *An. gambiae* in Port-Gentil [43], as predicted by the model, while Mouchet [42] did not record this species 26 years earlier. Elissa *et al*[44] also found low concentrations of *An. gambiae s.s.* in Haut-Ogooué, which was also predicted by the model. In the north-eastern part of Gabon it has not been possible to find any recent mosquito surveys, and it is therefore hard to conclude if the predicted absence of *An. gambiae* in this region is correct.

To evaluate the quality of the model with respect to classifying the presence and absence of the species the methodology described previously was used. Table 1 shows the mean absolute error for the four papers [1-3,5], expert opinion and this model. For reference, a MAE of 1 would be equivalent to completely wrong predictions, and 0 would be perfect. While the genetic algorithm of Levine [2] and the predictions based on satellite imagery by Rogers [1] show poor skill, the recent papers by Moffet *et al*[5] Sinka *et al*[3] are great improvements compared to those. Still, they have less skill than the expert opinion if comparing to the unweighed MAE. This model (OMaWa) has lower MEA than all the models included in this analysis, and including weights in the MEA makes it superior even to the expert opinion. The occurrence data suggest the expert opinion for *An. arabiensis* is wrong over West Africa and Southern Cameroon. A mosquito survey in Namibia, and north-eastern Gabon, would also clarify the present-day species composition in these countries.

**Table 1:** Mean absolute error species presence/absence (Weighted mean absolute error)

	**Model**	**MAE**
1	Levine	0.33 (NA)
2	Rogers	0.29 (NA)
3	Moffet	0.20 (0.07)
4	Expert Opinion	0.07 (NA)
5	Sinka	0.13 (0.05)
6	OMaWa	0.07 (0.01)

#### Relative fraction of each species, Madagascar (TC50)

**Figure 4:**
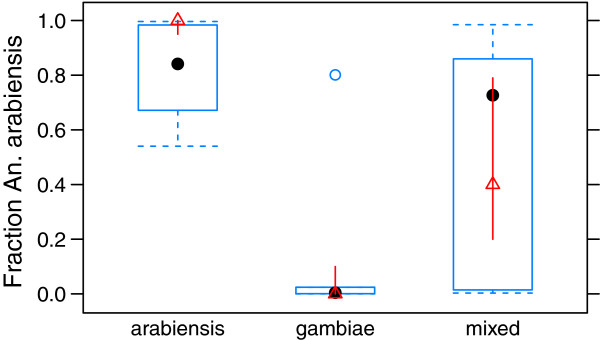
**Box-plot of fraction *An. arabiensis *, Madagascar. **Blue is the fraction from the model, while red is observations. The arabiensis group is where observations showed more than 85% *An. arabiensis*, gambiae is where observations showed less than 15% *An. arabiensis*, and mixed is the remaining data. Dot/triangle indicate the median.

Since Madagascar has a sharp separation between *An. arabiensis* and *An. gambiae s.s.*, the island is well suited to address whether the model is able to reproduce the relative fraction of each species.Three measures to evaluate the model was defined. For method a) the mean absolute error was 0.22. The box plot in Figure 4 show the fraction of *An. arabiensis* from the model, grouped by the fraction in the observations. It is clear, while capturing the main tendencies well, the model has problems with the exact separation between the two species. In the mixed group, the model tends to let one species dominate over the other, possibly letting *An. arabiensis* dominate too easily.

**Figure 5:**
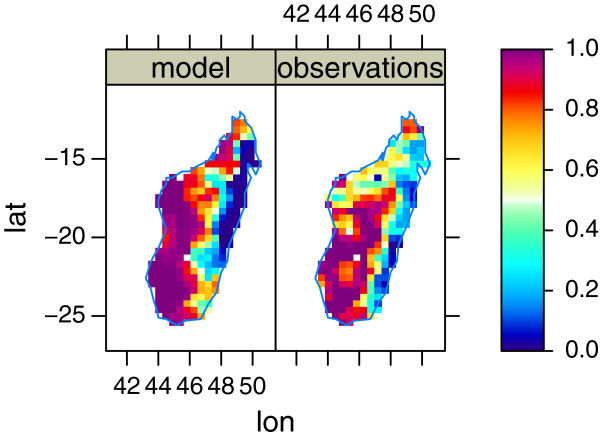
**Fraction of *An. arabiensis *. **Model 1990-2008, and observations smoothed with a squared inverse distance weighted kernel with cut-off at 100 km.

Figure 5, created using method b), shows the fraction of *An. arabiensis* as modelled, and observed. An eyeball comparison shows the separation is shifted westward in the model, and a bias in the South-Eastern tip of Madagascar. Whether this is a result of (climate) model resolution, failing to accurately separating the west/east gradient in topography, or the biological parametrization being inaccurate is hard to quantify. It is hoped this can be tested in a future analysis with higher model resolution.

Table 2 shows the distance to the closest model point, distance to the closest model point with correct prediction, and distance to the closest point with wrong prediction as described in c). At all quantiles the distance to the closest correct prediction is 1.5 to 7 smaller than the closest wrong prediction. A Mann-Whitney test with confidence level of 0.99 shows the difference in location between wrong and correct predictions is 9.84 (5.07 25.68) km (*p *< .0001). Thus, although with biases, it is concluded that distance to closest correct prediction and closest wrong prediction are non-identical populations.

**Table 2:** Distance to closest correct and wrong prediction

	**0%**	**10%**	**25%**	**50%**	**75%**	**90%**	**100%**
Distance to closest point	1.16	2.63	3.62	4.96	8.12	10.42	25.80
Distance to closest correct prediction	1.16	3.03	4.05	6.39	10.51	43.81	275.96
Distance to closest wrong prediction	1.63	4.06	5.55	28.05	73.72	112.66	311.81

Distance to the closest model point, distance to the closest model point with correct prediction, and distance to the closest point with wrong prediction. Model vs. observations.

### Temporal variability

It is important that mosquito models reproduce the seasonal cycle correctly, since this will be an indication of the sensitivity to climate. Here results from the model are compared to a number of observational studies. The comparison with each individual study might not have much information, but it is recommended that readers look at the results as a whole, having in mind the continental analysis showing the model is able to separate the distribution of *An. arabiensis* and *An. gambiae s.s.*. These results are meant to complement the continental analysis. Eth30 and Eth18 refers to the weather data used to drive OMaWa.

#### KEN11: 2007-2008 larva density in central Ethiopia (Eth30)

**Figure 6:**
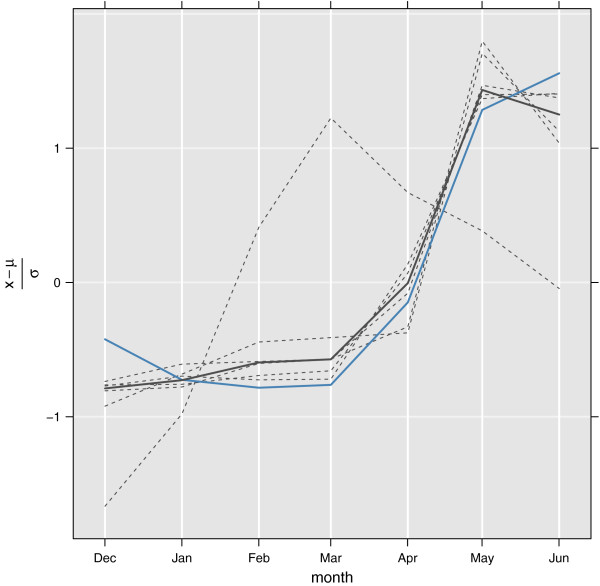
**Scaled variations over time of six locations (dashed grey line), and the median seasonality (solid grey line) in Central Ethiopia **[26] (data from KEN11). Blue solid line shows modelled relative seasonality in the same area.

In this study [26] Kenea *et al* reported the *An. arabiensis* larva density in six locations in central Ethiopia, December 2007 to June 2008. Five of the sites followed the same seasonality, while one had the highest density before the rainy season started. The model is not designed to capture such local variations, but is rather aiming to describe the median, or sometimes mean, state within a certain area. In their study all anopheline positive habitats present within a 500 m radius of each irrigated village/town and 700m along the major drainages (lake or river) were sampled. This means that the data should be comparable to what is modelled. The seasonality of larva density, $l_{\text{sum}}=\overset{4}{\underset{ı=1}{\sum }}l_{ı}$, per puddle area, *A*_*p*_, is then calculated as $C_{l}\frac{l_{\text{sum}}}{A_{p}[m^{2}]}$, where *C*_*l *_is a dimensionless constant. Correlations with the median relative seasonality, model vs. Kenea et al., is 0.97(0.81,0.99), and mean relative seasonality 0.92(0.55,0.98). The observations and modelled results can be seen in Figure 6.

#### TAY2006: 2001 mosquito catch Sille, Ethiopia (Eth30)

**Figure 7:**
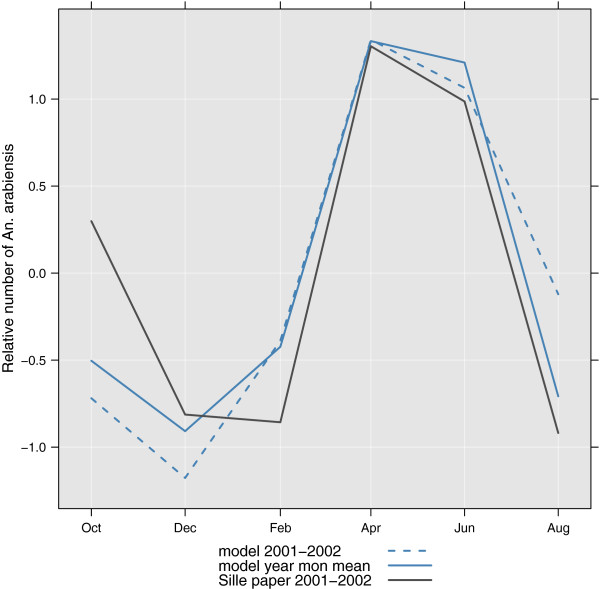
**Scaled variations over time of *An. arabiensis * in Sille, Ethiopia (data from TAY2006), observed (grey solid line), model 2001-2002 (solid blue line), model multi-year monthly mean (dashed blue line).**

In 2001-2002 Aseged Taye *et al*[27] recorded number of man biting *An. arabiensis* in Sille, Ethiopia. For simplicity it is assumed the human biting rate is independent of temperature and availability of breeding sites. This means the relative monthly mean sum of mosquitoes from the model should be directly comparable with the records from the paper. The model seems to under-predict the relative abundance of *An. arabiensis* in October 2001, and over-predict the rise in mosquito numbers in February. Otherwise the modelled number of mosquitoes seems comparable to what was observed by Taye *et al*. The correlation between observations and model (2001-2002) is 0.91(0.36,0.99). The observations and model results are shown in Figure 7.

#### YE2003: 2001 3 month larva variability in Zwai (Eth30)

**Figure 8:**
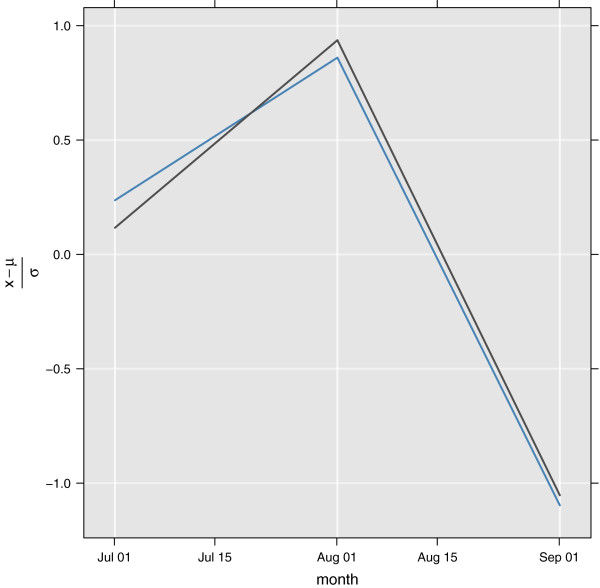
**Scaled variations over time of *An. arabiensis * larva in Zwai, Ethiopia (YE2003). **Observed (grey solid line), and model (solid blue line).

If it is assumed larva per dip has units $\text{LPD}=C\frac{\text{larva}}{m^{2}}$, where *C* is a constant, and that the samples are representative for a larger area, the relative number of larva in that area can be estimated as *LPD *· *W*_*a*_, where *W*_*a *_is the mean water area in *m*^2^. This way it is assumed the number of puddles is constant from July to September, and that the puddles only change their surface area. These values are roughly comparable to the modelled number of larva. Since only the latitude (and not the longitude) is reported in the paper, and Zwai is not located at latitude 9°*N*, model data between longitudes 38.69 to 39.23°*E* and latitudes 7.88 to 8.42°*N*, an area covering Zwai, were selected. Using this method correlation is 0.99(0.321.00). Confidence interval is estimated using 1,000 random samples of the points within the bounding box, and the 2.5*%* and 97.5*%* quantiles of the correlations is reported. Since the sample size is small and the data might not be directly comparable, the correlation should be interpreted with care. The data from the observations and the model can be seen in Figure 8.

#### BAL2001: 1999-2000 mosquito catch Awash, Ethiopia (Eth30)

**Figure 9:**
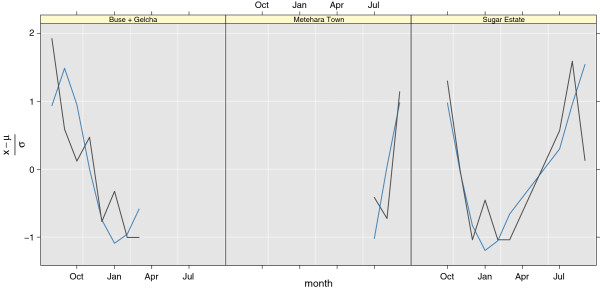
**Scaled variations over time of adult *An. arabiensis * in Awash, Ethiopia (BAL2001).** Observed (grey solid line), and model (solid blue line).

This study was carried out in 1999-2000 in Metehara at longitudes 39.50 to 40.00°*E* and latitudes 8.75 to 8.92°*N*. The data are based indoor space spray collections. Since the malaria model was not run for 1999, and 2000 is considered as a spin-up year, the multi-year monthly mean for the years 2001-2006, and 2008-2009 was used (since the climate model was done as two separate runs, one starting January 2000, and one starting January 2007). The observations are compared to the scaled sum of mosquitoes of all age groups, which should be comparable to what was reported in the thesis. Correlations in Buse + Gelcha (two locations described in the thesis) was 0.75(0.1,0.95), 0.79(0.27,0.95) for Sugar Estate, and 0.76 for Metehara Town. Confidence intervals are not reported for Metehara Town since the number of observations are low. The data can be seen in Figure 9.

#### FEK2012: 2009-2010 mosquito catch Chano Mille, Ethiopia (Eth18)

**Figure 10:**
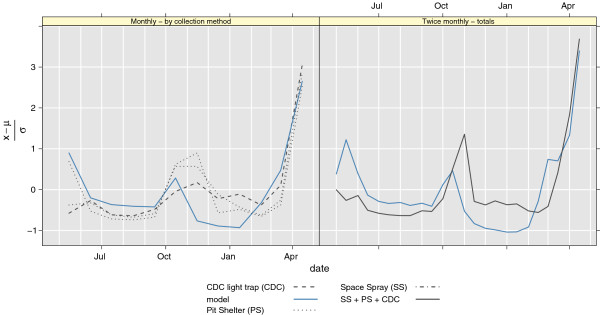
**Modeled and observed variations in *An. arabiensis *. **The left panel shows catches broken down to catch method (grey dotted lines), and modelled *An. arabiensis*. The right panel shows modelled mosquitoes and total twice monthly catches (data from FEK2012).

As seen in Figure 10, and correlations in Table 3, the model corresponds well with the observations in Chano, 2009-2010. While the weather station in Arba Minch recorded some heavy rainfall events in October/November 2009 the regional climate model did not capture these events, or did not dump the precipitation in the right location [45]. In general the driving model (WRF) was too wet in spring 2009, and too dry in autumn 2009. This might be the reason for the slight mismatch in mosquito numbers in these seasons. To have confidence in malaria/mosquito models at these fine scales, there is a need for a better representation of precipitation in the climate models. The differences between the trapping methods also highlight the uncertainty of related to data collection, especially when the number of mosquitoes is low. From December 2009 to March 2010 the observed number of *An. arabiensis* was very low (Figure 10). It is interesting that despite of this, malaria started to rise in these months [30].

**Table 3:** Correlations for model and mosquitoes captured in Chano Mille

	**Correlation**
Twice monthly	0.80 (0.58, 0.91)
Space Spray	0.83 (0.49, 0.95)
Pit Shelter	0.80 (0.43, 0.94)
CDC Light Trap	0.83 (0.48, 0.95)

Twice monthly shows correlation with total catch of *An. arabiensis* and modelled numbers. The Space Spray, Pit Shelter, and CDC Light Trap show correlation (95% confidence interval) with modelled number of mosquitoes and monthly catches using three different methods.

### Summary of temporal variability analysis

Each of the five case studies consist of short time series, with different observational methodologies. It was attempted to show how the model results can be compared to the different type of observations, and in general the model is in good agreement with the observations. Since none of the studies cover several years, it was only possible to validate whether the model captured the seasonal cycle in mosquito numbers. The good agreement with all of the five case studies, means the model probably responds correctly to the environment, and thus it is likely OMaWa can reproduce year-to-year variability as well.

## Conclusions

In this paper, the model has been validated using independent data. The model was designed to have a general validity, not being restricted to a specific locality. The study shows the model can capture the distribution and density of *An. gamibiae s.s.* and *An. arabiensis* across Africa, and that it is able to model the seasonal and year-to-year variations in mosquito densities. While the results are robust with respect to the mean distribution and density, there is a sampling bias related to the recent distribution in DR Congo, northern South Africa, southern Namibia, eastern Angola, Central African Republic, eastern Gabon, eastern Chad, South Sudan, and Somalia. This implies models can not be robustly validated in these regions, and that long term changes in the species composition can not be addressed. For the temporal variability, the model has only been validated for Ethiopia, using short time series. Although the model matches well with the observations, most of the time series are short, implying the ability to reproduce year-to-year variations has not been fully addressed.

The results suggest sufficiently high bovine density influences the large-scale distribution of *An. arabiensis*. Similarly, the presence of *An. gambiae s.s.* is linked to the presence of humans, modulated by the density of *An. arabiensis*. Water and air temperature, and availability of breeding sites play secondary roles for the continental distribution of these species, but might be locally important in margin zones. The recent distribution shifts in species composition observed in Kenya [13,46] might be partially explained by increased mortality of *An. gambiae s.s.* due to interventions like IRS and LLINs. An alternative explanation might be the competitive advantage of *An. arabiensis* efficiently feeding on cattle, and thus suppressing the number of *An. gambiae s.s.* through easier access to blood, and thus reproducing at a higher rate. Over time, these interventions mainly reduce the human biting rate, and not necessarily the longevity of mosquitoes; the most efficient measure in MacDonald’s formula of the basic reproductive number. Next, it can be challenged if a reduction of the number of breeding sites, lowering the number of adult mosquitoes per human, would be as efficient, and cost-effective, as IRS and LLINs over time. Studies on the long-term effect of interventions on the mortality rate of mosquitoes is needed to evaluate how these interventions work in practice. The large scale distribution of *An. arabiensis*, and its relation to cattle distribution, also rises the question of this species is using the odour of bovine to navigate, and if this causes of the observed coexistence of *An. arabiensis* and cattle. If this applies on large scales, there are reasons to believe the same mechanisms manifest themselves on small scales. In that case, keeping cattle separate from humans should further reduce the human biting rate in areas where *An. arabiensis* is the dominant species.

Several studies have found out the gene flow of *An. gambiae s.s.* and *An. arabiensis*, and how the species have spread, and is evolving, across Africa. In the model, which gives a good representation of the distribution of the two species, *An. gambiae s.s.* is spreading most efficient on surfaces with continuous human populations, while *An. arabiensis* disperse more easily on surfaces with continuous cattle populations. It is hypothesized the lack of such a human surface between Kenya and Ethiopia can explain the absence of *An. gambiae s.s.* in Ethiopia; - to spread to Ethiopia, there is a need of a more or less continuous human population cover from Lake Victoria to southern Ethiopia, sufficient breeding sites, and temperatures which are not too extreme. Thus, not only climate control the presence and absence of these species, but also the availability of hosts. This has implications for the ability to project the future distribution of the two species.

Before models can be related to improving human health, or guide which interventions are to be applied where, there is a need to understand the system of interest. Validation is an important part of this process. Concluding based on too little data, and basing projections of for example the effects of climate change on models which have not been validated, is dangerous [47], might mislead the public, and lead to less confidence in science. The way forward would be to include effects on interventions. This would allow us to understand how residual spraying and bed nets influence the mosquito populations, and in turn malaria. Incorporating interventions in a continental model requires a) spatial data describing which interventions were applied when, and b) the long term effect of these interventions. Currently such data might exist, but have not been systematized for use by the research community. In these two papers, the focus has deliberately been on the mosquito population. By looking at each component involved in malaria transmission separately, the understanding of the dynamics of malaria can be improved. This process is crucial to robustly estimate how a changing environment and society, has changed, and will change, the premises for malaria transmission.

## Competing interests

The authors declare that they have no competing interests.

## Authors’ contributions

The work presented here was carried out in collaboration between all authors. BL and AS defined the research theme. TML designed methods and mosquito experiments, did the model runs, analysed the data, interpreted the results and wrote the paper. DK, AS and TML designed the regional climate simulations, and evaluated those. FM, MB and TGM collected data for validation, and contributed with comments on the biology of *An. gambiae s.l.*. All authors have contributed to, seen and approved the manuscript.

## References

[B1] Rogers DJ Randolph SE Snow RW Hay SI Satellite imagery in the study and forecast of malaria Nature 2002 415 710 715 [http://www.ncbi.nlm.nih.gov/pubmed/11832960] 10.1038/415710a 11832960 PMC3160466

[B2] Levine RS Peterson AT Benedict MQ Geographic and ecologic distributions of the Anopheles gambiae complex predicted using a genetic algorithm Am J Trop Med Hyg 2004 70 105 109 [http://www.ncbi.nlm.nih.gov/pubmed/14993618] 14993618

[B3] Sinka ME Bangs MJ Manguin S Coetzee M Mbogo CM Hemingway J Patil AP Temperley WH Gething PW Kabaria CW Okara RM Van Boeckel T Godfray HCJ Harbach RE Hay SI The dominant Anopheles vectors of human malaria in Africa, Europe and the Middle East: occurrence data, distribution maps and bionomic precis Parasit Vectors 2010 3 117 [http://www.ncbi.nlm.nih.gov/pubmed/21129198] 10.1186/1756-3305-3-117 21129198 PMC3016360

[B4] Lindsay SW Parson L Thomas CJ Mapping the ranges and relative abundance of the two principal African malaria vectors, Anopheles gambiae sensu stricto and An. arabiensis, using climate data Proc Biol Sci 1998 265 847 854 [http://www.ncbi.nlm.nih.gov/pubmed/9633110] 10.1098/rspb.1998.0369 9633110 PMC1689061

[B5] Moffett A Shackelford N Sarkar S Malaria in Africa: vector species’ niche models and relative risk maps PLoS One 2007 2 e824 [http://www.ncbi.nlm.nih.gov/pubmed/17786196] 10.1371/journal.pone.0000824 17786196 PMC1950570

[B6] MacDonald G The Epidemiology and Control of Malaria 1957 London: Oxford University Press

[B7] Pluess B Tanser FC Lengeler C Sharp BL Indoor residual spraying for preventing malaria Cochrane Database Syst Rev 2010 CD006657 [http://www.ncbi.nlm.nih.gov/pubmed/20393950] 20393950 10.1002/14651858.CD006657.pub2 PMC6532743

[B8] Noor AM Mutheu JJ Tatem AJ Hay SI Snow RW Insecticide-treated net coverage in Africa: mapping progress in 2000-07 Lancet 2009 373 58 67 [http://www.ncbi.nlm.nih.gov/pubmed/19019422] 10.1016/S0140-6736(08)61596-2 19019422 PMC2652031

[B9] Coluzzi M Petrarca V Dideco M Chromosomal inversion intergradation and incipient speciation in Anopheles gambiae B Zool 1985 52 45 63 10.1080/11250008509440343

[B10] della Torre A Tu Z Petrarca V On the distribution and genetic differentiation of Anopheles gambiae s.s. molecular forms Insect Biochem Mol Biol 2005 35 755 769 [http://www.ncbi.nlm.nih.gov/pubmed/15894192] 10.1016/j.ibmb.2005.02.006 15894192

[B11] Favia G della Torre A Bagayoko M Lanfrancotti A Sagnon N Touré YT Coluzzi M Molecular identification of sympatric chromosomal forms of Anopheles gambiae and further evidence of their reproductive isolation Insect Mol Biol 1997 6 377 383 [http://www.ncbi.nlm.nih.gov/pubmed/9359579] 10.1046/j.1365-2583.1997.00189.x 9359579

[B12] Coetzee M Distribution of the African malaria vectors of the Anopheles gambiae complex Am J Trop Med Hyg 2004 70 103 104 [http://www.ncbi.nlm.nih.gov/pubmed/14993617] 14993617

[B13] Bayoh MN Mathias DK Odiere MR Mutuku FM Kamau L Gimnig JE Vulule JM Hawley WA Hamel MJ Walker ED Anopheles gambiae: historical population decline associated with regional distribution of insecticide-treated bed nets in western Nyanza Province, Kenya Malar J 2010 9 62 [http://www.ncbi.nlm.nih.gov/pubmed/20187956] 10.1186/1475-2875-9-62 20187956 PMC2838909

[B14] Adamou A Dao A Timbine S Kassogue Y Yaro AS Diallo M Traore SF Huestis DL Lehmann T The contribution of aestivating mosquitoes to the persistence of Anopheles gambiae in the Sahel Malar J 2011 10 151 [http://www.ncbi.nlm.nih.gov/pubmed/21645385] 10.1186/1475-2875-10-151 21645385 PMC3123247

[B15] Derua YA Alifrangis M Hosea KM Meyrowitsch DW Magesa SM Pedersen EM Simonsen PE Change in composition of the Anopheles gambiae complex and its possible implications for the transmission of malaria and lymphatic filariasis in north-eastern Tanzania Malar J 2012 11 188 [http://www.ncbi.nlm.nih.gov/pubmed/22681999] 10.1186/1475-2875-11-188 22681999 PMC3469399

[B16] Lunde TM Korecha D Loha E Sorteberg A Lindtjørn B A dynamic model of some malaria-transmitting anopheline mosquitoes of the Afrotropical region. I. Model description and sensitivity analysis Malar J 2013 12 28 [http://www.ncbi.nlm.nih.gov/pubmed/23342980] 10.1186/1475-2875-12-28 23342980 PMC3664083

[B17] Ermert V Fink AH Jones AE Morse AP Development of a new version of the Liverpool Malaria Model. II. Calibration and validation for West Africa Malar J 2011 10 62 [http://www.ncbi.nlm.nih.gov/pubmed/21410939] 10.1186/1475-2875-10-62 21410939 PMC3070689

[B18] OMaWa [http://uib.no/pub/gfi/tlu004/OMaWa/]

[B19] Coetzee M Craig M le Sueur D Mapping the distribution of members of the Anopheles gambiae complex in Africa and adjacent islands Parasitol Today 2000 16 74 77 10.1016/S0169-4758(99)01563-X 10652493

[B20] Biodiversity occurrence data published by: Walter Reed Biosystematics Unit Smithsonian Institution GBIF Data Portal [http://data.gbif.org]

[B21] Hay SI Sinka ME Okara RM Kabaria CW Mbithi PM Tago CC Benz D Gething PW Howes RE Patil AP Temperley WH Bangs MJ Chareonviriyaphap T Elyazar IRF Harbach RE HemingwayM J Manguin S Mbogo CM Rubio-Palis Y Godfray HCJ Developing global maps of the dominant Anopheles vectors of human malaria PLoS Med 2010 7 e1000209 [http://www.ncbi.nlm.nih.gov/pubmed/20161718] 10.1371/journal.pmed.1000209 PMC2817710 20161718

[B22] Jack R Dubinin M Massing M Luthman L Georeferencer GDAL. Tech. rep. http://www.gdal.org/;2011. [http://gis-lab.info/qa/qgis-georef-new-eng.html]

[B23] Willmott CJ Matsuura K Advantages of the mean absolute error (MAE) over the root mean square error (RMSE) in assessing average model performance Clim Res 2005 30 79 82 [http://www.int-res.com/abstracts/cr/v30/n1/p79-82/]. [10.3354/cr030079].

[B24] Pock Tsy JML Duchemin JB Marrama L Rabarison P Le Goff G Rajaonarivelo V Robert V Distribution of the species of the Anopheles gambiae complex and first evidence of Anopheles merus as a malaria vector in Madagascar Malar J 2003 2 33 [http://www.ncbi.nlm.nih.gov/pubmed/14609436] 10.1186/1475-2875-2-33 14609436 PMC269986

[B25] Chauvet G Répartition et écologie du complex Anopheles gambiae à Madagascar Cah ORSTOM sér Ent Méd 1969 VII 235 278

[B26] Kenea O Balkew M Gebre-Michael T Environmental factors associated with larval habitats of anopheline mosquitoes (Diptera: Culicidae) in irrigation and major drainage areas in the middle course of the Rift Valley, central Ethiopia J Vector Borne Dis 2011 48 85 92 [http://www.ncbi.nlm.nih.gov/pubmed/21715730] 21715730

[B27] Taye A Hadis M Adugna N Tilahun D Wirtz RA Biting behavior and Plasmodium infection rates of Anopheles arabiensis from Sille, Ethiopia Acta Trop 2006 97 50 54 [http://www.ncbi.nlm.nih.gov/pubmed/16171769] 10.1016/j.actatropica.2005.08.002 16171769

[B28] Ye-Ebiyo Y Pollack RJ Kiszewski A Spielman A Enhancement of development of larval Anopheles arabiensis by proximity to flowering maize (Zea mays) in turbid water and when crowded Am J Trop Med Hyg 2003 68 748 752 [http://www.ncbi.nlm.nih.gov/pubmed/12887038] 12887038

[B29] Balkew M Studies on the anopheline mosquitoes of Metehara and surrounding areas in relation to malaria transmission *Master’s thesis: *Department of Biology, Addis Ababa University; 2001.

[B30] Loha E Lindtjorn B Predictors of Plasmodium falciparum Malaria incidence in Chano Mille, South Ethiopia: a longitudinal study Am J Trop Med Hyg 2012 87 450 459 [http://www.ncbi.nlm.nih.gov/pubmed/22826493] 10.4269/ajtmh.2012.12-0155 22826493 PMC3435347

[B31] Massebo F Balkew M Gebre-Michael T Lindtjørn B Blood meal origins and insecticide susceptibility of Anopheles arabiensis from Chano in South-West Ethiopia Parasit Vectors 2013 6 44 10.1186/1756-3305-6-44 23433306 PMC3606335

[B32] Skamarock WC Klemp JB Dudhia J Gill DO Barker DM Wang W Powers JG A description of the advanced research WRF version 2. 2005 Tech. rep., The National Center for Atmospheric Research

[B33] Kain J Fritsch J Convective parameterization for mesoscale models: The Kain–Fritsch scheme Representation Cumulus Convection Numerical Models Metor Monogr, Am Meteor Soc 1993 24 165 170

[B34] Kain J The Kain–Fritsch convective parameterization: An update J Appl Meteorology 2004 43 170 181 10.1175/1520-0450(2004)043<0170:TKCPAU>2.0.CO;2

[B35] Zhang C Wang Y Hamilton K Improved Representation of Boundary Layer Clouds over the Southeast Pacific in ARW-WRF Using a Modified Tiedtke Cumulus Parameterization Scheme Monthly Weather Rev 2011 139 3489 3513 10.1175/MWR-D-10-05091.1

[B36] Tiedtke M A comprehensive mass flux scheme for cumulus parameterization in large-scale models Monthly Weather Rev 1989 117 1779 1800 10.1175/1520-0493(1989)117<1779:ACMFSF>2.0.CO;2

[B37] Kerah-Hinzoumbe C Peka M Nwane P Donan-Gouni I Etang J Same-Ekobo A Simard F Insecticide resistance in Anopheles gambiae from south-western Chad, Central Africa Malar J 2008 7 192 [http://www.ncbi.nlm.nih.gov/pubmed/18823537] 10.1186/1475-2875-7-192 18823537 PMC2566574

[B38] Brink C Malaria control in the Northern Transvaal S Afr Med J 1958 32 800 809 13580479

[B39] Meillon BD Malaria survey of South-West Africa Bull World Health Organ 1951 4 333 417 14886720 PMC2554078

[B40] Service MW Contribution to the knowledge of the mosquitoes (Diptera, Culicdae) of Gabon Cah ORSTOM sér Ent Méd 1976 3 259 263

[B41] Sylla EH Kun JF Kremsner PG Mosquito distribution and entomological inoculation rates in three malaria-endemic areas in Gabon Trans R Soc Trop Med Hyg 2000 94 652 656 [http://www.ncbi.nlm.nih.gov/pubmed/11198649] 10.1016/S0035-9203(00)90219-0 11198649

[B42] Mouchet J Survey of Potential Yellow Fever Vectors in Gabon and Chad World Health Organ 1970 VBC71.279 1 10

[B43] Mourou JR Coffinet T Jarjaval F Pradines B Amalvict R Rogier C Kombila M Pages F Malaria transmission and insecticide resistance of Anopheles gambiae in Libreville and Port-Gentil, Gabon Malar J 2010 9 321 [http://www.ncbi.nlm.nih.gov/pubmed/21070655] 10.1186/1475-2875-9-321 21070655 PMC2995799

[B44] Elissa N Karch S Bureau P Ollomo B Lawoko M Yangari P Ebang B Georges AJ Malaria transmission in a region of savanna-forest mosaic, Haut-Ogooué, Gabon J Am Mosq Control Assoc 1999 15 15 23 [http://www.ncbi.nlm.nih.gov/pubmed/10342264] 10342264

[B45] Stensrud D Parameterization Schemes: Keys to Understanding Numerical Weather Prediction Models 2007 Cambridge University Press [http://books.google.no/books?id=lMXSpRwKNO8C]

[B46] Mwangangi JM Mbogo CM Orindi BO Muturi EJ Midega JT Nzovu J Gatakaa H Githure J Borgemeister C Keating J Beier JC Shifts in malaria vector species composition and transmission dynamics along the Kenyan coast over the past 20 years Malar J 2013 12 13 [http://www.ncbi.nlm.nih.gov/pubmed/23297732] 10.1186/1475-2875-12-13 23297732 PMC3544599

[B47] Lunde TM Bayoh MN Lindtjørn B How malaria models relate temperature to malaria transmission Parasit Vectors 2013 6 20 [http://www.ncbi.nlm.nih.gov/pubmed/23332015] 10.1186/1756-3305-6-20 23332015 PMC3598736

